# A Case of Ovarian Actinomycosis

**DOI:** 10.1080/10647440300025517

**Published:** 2003

**Authors:** Masahiro Iwasaki, Akira Nishikawa, Noriyuki Akutagawa, Takashi Fujimoto, Mizue Teramoto, Ryuichi Kudo

**Affiliations:** Department of Obstetrics and Gynecology School of Medicine Sapporo Medical University S1W16, Chuo-ku Sapporo 060-8543 Japan

## Abstract

*Background:*  Pelvic actinomycosis is uncommon and often presents as a complication of an intrauterine device (IUD). A diagnosis of actinomycosis can be made from the finding of sulfur granules within inflammatory exudate on histologic examination after surgery. However, it may be possible to diagnose actinomycosis before surgery by finding *Actinomyces*-like organisms on Papanicolaou smears.

*Case:* A 41-year-old woman had been diagnosed as having a pelvic abscess, and bilateral salpingo-oophorectomy was performed. She had been an IUD user for 6 years. *Actinomyces*-like organisms were detected in her previous Papanicolaou cervical smears. If the patient had been treated when the *Actinomyces*-like organisms were detected by Papanicolaou smears, the serious ovarian actinomycosis might have been avoided.

*Conclusion:* We suggest that routine cervical examinations are important for women who are IUD users.


*Infect Dis Obstet Gynecol 2003;11:171–173*

### A case of ovarian actinomycosis

**Masahiro Iwasaki, Akira Nishikawa, Noriyuki Akutagawa, Takashi Fujimoto, Mizue Teramoto and Ryuichi Kudo**

*Department of Obstetrics and Gynecology, School of Medicine, Sapporo Medical University, Sapporo, Japan*

***Background:*** Pelvic actinomycosis is uncommon and often presents as a complication of an intrauterine device (IUD). A diagnosis of actinomycosis can be made from the finding of sulfur granules within inflammatory exudate on histologic examination after surgery. However, it may be possible to diagnose actinomycosis before surgery by finding *Actinomyces*-like organisms on Papanicolaou smears.

***Case:*** A 41-year-old woman had been diagnosed as having a pelvic abscess, and bilateral salpingo-oophorectomy was performed. She had been an IUD user for 6 years. *Actinomyces*-like organisms were detected in her previous Papanicolaou cervical smears. If the patient had been treated when the *Actinomyces*-like organisms were detected by Papanicolaou smears, the serious ovarian actinomycosis might have been avoided.

***Conclusion:*** We suggest that routine cervical examinations are important for women who are IUD users.

Key words: INTRAUTERINE DEVICE; PAPANICOLAOU SMEARS; SULFUR GRANULES

Pelvic actinomycosis is uncommon and often presents as a complication of an intrauterine device (IUD). Almost 85% of cases occur in women who have had an IUD in place for 3 or more years[1]. The diagnosis of actinomycosis can be confirmed by culture. However, it is often difficult to culture *Actinomyces*. In fact, the detection rate of *Actinomyces* in patients with pelvic actinomycosis is as low as 2%[2]. Therefore a diagnosis of actinomycosis can be made from the finding of sulfur granules within inflammatory exudate on histologic examination after surgery. However, it may be possible to diagnose actinomycosis before surgery by the finding of *Actinomyces*-like organisms on Papanicolaou smears.

We describe a case of rare ovarian actinomycosis associated with an IUD, and cytological findings that were obtained 2 and 4 years before the onset of the disease.

### CASE

A 41-year-old woman, gravida 4, para 3, was admitted to hospital due to lower abdominal pain and a history of high fever for 4 days. Physical examination revealed tenderness in the lower abdomen. A computed tomography (CT) scan and pelvic ultrasound examination revealed bilateral adnexal tumors with irregular margins. Laboratory tests demonstrated marked leukocytosis (white blood cell count 23 800/mm$^3$) and elevated C-reactive protein concentration (27.8 mg/dl). CA125 and CA19-9 were within the normal range. The patient had been an IUD user for 6 years. She had undergone a Cesarean section (at 23 years of age) and an appendectomy (at 25 years). She did not have any history of sexually transmitted disease, and her serological test was HIV-negative. We diagnosed her condition

Correspondence to: Masahiro Iwasaki, MD, Department of Obstetrics and Gynecology, School of Medicine, Sapporo Medical University, S1W16, Chuo-ku, Sapporo 060-8543, Japan. Email: miwasaki@sapmed.ac.jp

© 2003 The Parthenon Publishing Group as pelvic abscess, and bilateral salpingo-oophorectomy was performed.

At the time of surgery, the bilateral ovaries and the Fallopian tubes formed an irregular mass and were covered with a film-like adhesion. Both ovaries were enlarged, and the dimensions of the right and left ovaries were 7 × 6 cm and 7 × 4 cm, respectively. They were mostly replaced by soft, gray-yellow tissue with extensive degeneration. They had no abscess cavity. There was adhesion between the intestines in the pelvic wall due to the peritonitis.

Histological findings revealed multiple abscesses that consisted of neutrophils and foamy histiocytes in both ovaries and Fallopian tubes. Figure 1 shows the microscopic examination of the right adnexa. The characteristic actinomycotic granules (sulfur granules) were seen in the purulent exudate of the right ovary.

We reviewed the patient's previous cervical smears obtained at routine check-ups taken by the doctor who treated the patient 2 and 4 years before the surgery (i.e. 4 and 2 years after IUD insertion, respectively). Figure 2 shows a cervical smear taken 2 years before surgery, with a colony of *Actinomyces*-like organisms.

Postoperatively, the patient was treated with intravenous piperacillin and tetracycline for 1 week and then oral penicillin for 4 weeks, and her course was uneventful. She currently receives daily doses of conjugated estrogen (0.625 mg) plus medroxyprogesterone acetate (2.5 mg) for hormone replacement therapy.

### CONCLUSION

Actinomycosis is a chronic suppurative and granulomatous bacterial infection caused by *Actinomyces israelii*, which is a Gram-positive, non-spore-forming anaerobic or microaerophilic organism. *Actinomyces* species are normally found in the human oropharynx, gastrointestinal tract and vagina, and they grow slowly under conditions of reduced oxygen. Pelvic actinomycosis is uncommon, but there have been many reports because of the difficulty of the diagnosis. The ascending route from the lower genital tract is thought to be important for the infection[3]. The common symptoms of pelvic actinomycosis are abdominal pain, an abdominal mass, fever and weight loss. It is often misdiagnosed as a gynecological malignancy[4–6]. Ovarian actinomycosis is rarer, because the structure of the ovary is resistant to surrounding inflammatory disease[4]. It has been assumed that bacteria enter the ovary when its surface is broken by the process of ovulation. Treatment of actinomycosis consists of long-term antibiotic therapy and adequate surgery, such as drainage of the abscess and reduction of infected tissue. For antibiotic therapy of actinomycosis, oral penicillin and tetracycline for 6–12 months are effective. If the focus is removed sufficiently by

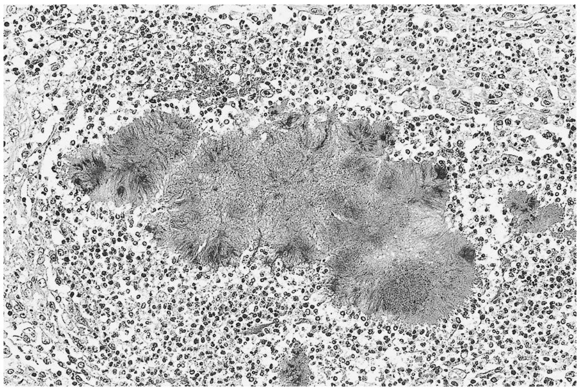

**Figure 1** Microscopic examination reveals inflammatory reaction in the right ovary with abscess formation. Colonies of *Actinomyces* (sulfur granules) are present within a purulent exudation (hematoxylin and eosin, × 170)

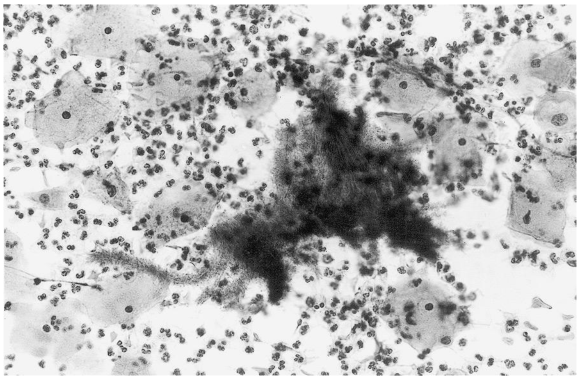

**Figure 2** Cervical Papanicolaou smears show a colony of *Actinomyces*-like organisms surrounded by neutrophils. Filaments are visible around the periphery of the colony (Papanicolaou, × 340)

surgery, antibiotic therapy may be finished within a shorter period. A relatively short course of antibiotic treatment was successful after surgical removal in our patient. It is well known that IUD users are at risk for pelvic actinomycosis[1–3,5–11]. An IUD that is placed for a long period causes an inflammatory response on the surface of the endometrium[2,3,7]. Papanicolaou smears are useful for the evaluation of patients with pelvic actinomycosis associated with an IUD[2,6,8,10]. Some researchers have reported that 53–80% of women who had *Actinomyces* on Papanicolaou smears actually had symptoms[10,11]. A diagnosis of actinomycosis may be made before surgery in a few cases by finding *Actinomyces*-like organisms on Papanicolaou smears. Burkman and colleagues[8] reported that women for whom *Actinomyces* is present on Papanicolaou smears have a 3.6-fold increased risk of hospitalization for pelvic inflammatory disease (PID) compared with women without *Actinomyces*. We could not diagnose our patient's actinomycosis before the onset of the disease. However, her previous Papanicolaou smears revealed *Actinomyces*-like organisms. If the patient had been treated when these organisms were detected by Papanicolaou smears, the serious ovarian actinomycosis might have been avoided. Lippes[9] reported in a review that the detection of *Actinomyces* by Papanicolaou smears could not be used to diagnose actinomycosis, and even that the IUD of a patient with a positive culture of *Actinomyces* does not require removal. However, many authors have advocated the removal of any IUD if *Actinomyces*-like organisms are detected by cervical smear[2,5,6,8,11]. Furthermore, it has been proposed that the clearance of organisms should be checked by a 6-week repeat smear. The IUD might then be reinserted in the patient on confirmation of the absence of *Actinomyces*-like organisms.

### REFERENCES

1. Schmidt WA. IUDs, inflammation and infection: assessment after two decades of IUD use. *Hum Pathol* 1982;13:878–81
2. Hager WD, Douglas B, Majmudar B, *et al.* Pelvic colonization with *Actinomyces* in women using intrauterine contraceptive devices. *Am J Obstet Gynecol* 1979;135:680–4
3. Henderson SR. Pelvic actinomycosis associated with an intrauterine device. *Obstet Gynecol* 1973; 41:726–32
4. Koshiyama M, Yoshida M, Fujii H, *et al.* Ovarian actinomycosis complicated by diabetes mellitus simulating an advanced ovarian carcinoma. *Eur J Obstet Gynecol Reprod Biol* 1999;87:95–9
5. Muller-Holzner E, Ruth NR, Abfalter E, *et al.* IUD-associated pelvic actinomycosis: a report of five cases. *Int J Gynecol Pathol* 1995;14:70–4
6. Fiorino AS. Intrauterine contraceptive device-associated actinomycotic abscess and *Actinomyces* detection on cervical smear. *Obstet Gynecol* 1996;87:142–9
7. Gupta PK, Malkani PD, Bhasin K. Cellular response in the uterine cavity after IUD insertion and structural changes of the IUD. *Contraception* 1971;4:375–84
8. Burkman R, Schlesselman S, McCaffrey L, *et al.* The relationship of genital tract actinomycetes and the development of pelvic inflammatory disease. *Am J Obstet Gynecol* 1982;143:585–9
9. Lippes J. Pelvic actinomycosis: a review and preliminary look at prevalence. *Am J Obstet Gynecol* 1999;180:265–9
10. Bhagavan BS, Gupta PK. Genital actinomycosis and intrauterine contraceptive devices. Cytopathologic diagnosis and clinical significance. *Hum Pathol* 1978;9:567–78
11. Spence MR, Gupta PK, Frost JK, *et al.* Cytologic detection and clinical significance of *Actinomyces israelii* in women using intrauterine contraceptive devices. *Am J Obstet Gynecol* 1978;131:295–8

RECEIVED 11/29/02; ACCEPTED 05/27/03
